# Geographic clusters of objectively measured physical activity and the characteristics of their built environment in a Swiss urban area

**DOI:** 10.1371/journal.pone.0252255

**Published:** 2022-02-23

**Authors:** Juan R. Vallarta-Robledo, Stéphane Joost, Marco André Vieira Ruas, Cédric Gubelmann, Peter Vollenweider, Pedro Marques-Vidal, Idris Guessous

**Affiliations:** 1 Division and Department of Primary Care Medicine, Geneva University Hospitals, Geneva, Switzerland; 2 Faculty of Medicine, University of Geneva, Geneva, Switzerland; 3 Group of Geographic Information Research and Analysis in Population Health (GIRAPH), Geneva, Switzerland; 4 Laboratory of Geographic Information Systems (LASIG), School of Architecture, Civil and Environmental Engineering (ENAC), École Polytechnique Fédérale de Lausanne (EPFL), Lausanne, Switzerland; 5 La Source, School of Nursing, University of Applied Sciences and Arts Western Switzerland (HES-SO), Lausanne, Switzerland; 6 Department of Medicine, Internal Medicine, Lausanne University Hospital (CHUV) and University of Lausanne, Lausanne, Switzerland; Peking University Shenzhen Graduate School, CHINA

## Abstract

**Introduction:**

Evidence suggests that the built environment can influence the intensity of physical activity. However, despite the importance of the geographic context, most of the studies do not consider the spatial framework of this association. We aimed to assess individual spatial dependence of objectively measured moderate and vigorous physical activity (MVPA) and describe the characteristics of the built environment among spatial clusters of MVPA.

**Methods:**

Cross-sectional data from the second follow-up (2014–2017) of CoLaus|PsyCoLaus, a longitudinal population-based study of the Lausanne area (Switzerland), was used to objectively measure MVPA using accelerometers. Local Moran’s I was used to assess the spatial dependence of MVPA and detect geographic clusters of low and high MVPA. Additionally, the characteristics of the built environment observed in the clusters based on raw MVPA and MVPA adjusted for socioeconomic and demographic factors were compared.

**Results:**

Data from 1,889 participants (median age 63, 55% women) were used. The geographic distribution of MVPA and the characteristics of the built environment among clusters were similar for raw and adjusted MVPA. In the adjusted model, we found a low concentration of individuals within spatial clusters of high MVPA (median: 38.5mins; 3% of the studied population) and low MVPA (median: 10.9 mins; 2% of the studied population). Yet, clear differences were found in both models between clusters regarding the built environment; high MVPA clusters were located in areas where specific compositions of the built environment favor physical activity.

**Conclusions:**

Our results suggest the built environment may influence local spatial patterns of MVPA independently of socioeconomic and demographic factors. Interventions in the built environment should be considered to promote physically active behaviors in urban areas.

## Introduction

Regular physical activity improves health status and prevents chronic diseases [1, 2]. For such reasons, the World Health Organization (WHO) recommends 150 minutes of moderate-intensity, 75 minutes of vigorous-intensity per week, or a combination of moderate and vigorous physical activity (MVPA) [3]. However, despite the benefits of consistent MVPA, current trends do not show an encouraging increment of these recommendations in the population [4]. Regular practice of physical activity depends on a series of determinants, including biological, psychological, cultural, socioeconomic, and environmental [5, 6].

The built environment can influence physical activity and promote healthy lifestyles through different settings [7, 8]. This suggests human behaviors are not only responsive to individual factors (i.e. socioeconomic, demographic, and attitudinal) but also, to the contextual characteristics of the built environment [9] that can provide high degrees of motivation and satisfaction [10]. Ewing & Cervero [11] claim five built environmental contextual elements affect physically active behaviors: density (e.g. population), diversity (e.g. land use mix), design (e.g. street connectivity), destination accessibility (e.g. ease of travel to the desired destination), and distance to transit (e.g. accessibility to public transport). Indeed, evidence states that individuals living in areas with higher accessibility to walking, cycling, and public transport infrastructure, street connectivity and accessibility to diverse destinations, the presence of diverse land use (i.e. residential zones mixed with public, recreational and commercial areas), larger access to recreational areas of quality, and greater population density have been associated with higher levels of physical activity [12–14].

This is of major importance as it supports the development of public policies promoting physically active behaviors at the community level. To facilitate healthy environments and target areas for intervention, it is important to identify and understand the spatial distribution of health outcomes and environmental characteristics [15, 16]. Spatial statistics and related clustering methods are of great help to reveal non-random geographic patterns of a given outcome and have become popular in recent years in epidemiological studies due to the availability of high-resolution spatial data [17, 18]. Spatial approaches have proven to be more reliable for assessing the contextual effect of the neighborhood environment on health outcomes than traditional multilevel methods since the formers consider distance space as continuum rather than assuming space is invariant and based on large geographic units [19, 20]. Furthermore, spatial clustering methods allow the identification of geographic areas at risk, which may be helpful for policymakers to target and monitor local areas of intervention.

However, despite the importance of the geographic context in the association between the built environment and physical activity, most previous research did not consider the spatial factor. Studies using a spatial methodology to assess this relationship have shown partial associations [21–28]. Some studies agree that a higher population density, land use mix, and street connectivity promote physical active behaviors [23, 26], while others have found weak [27] or partial associations (the built environment was associated with higher physical activity levels only in certain counties or urban areas) [21, 22, 24, 25, 28]. This mixed evidence may result from the fact that these works analyze the association at an aggregated level (i.e. statistical subsectors, counties, etc.) [21–25], which may not always reflect the real geographic context [29, 30] as it is very likely that local differences in the composition of the built environment exist. Furthermore, of these studies, only one objectively measured physical activity [23]. Among the research works assessing spatial patterns of physical activity at an individual scale, all were based on self-reported physical activity [26–28], which limits their conclusions.

We aimed to assess the spatial dependence of objectively measured MVPA in the urban area of Lausanne using individual accelerometry and geolocated data. Additionally, we assessed and compared the built environment characteristics among the spatial clusters of both raw MVPA and MVPA adjusted for socioeconomic and demographic factors.

## Materials and methods

### Urban area of Lausanne

The city of Lausanne covers an area of 41.37 km^2^ with a population of 144,790 inhabitants in 2017, and it is divided into 81 statistical sub-sectors (https://www.lausanne.ch/officiel/statistique.html). It has around 350 hectares of parks and gardens, 111 km of cycling pathways (https://www.lausanne.ch/officiel/statistique.html), two metro and 42 bus and trolleybus lines (https://rapportannuel.t-l.ch) (S1 Fig).

### Health data

Data were obtained from CoLaus|PsyCoLaus cohort, a longitudinal population-based study started in 2003–2006, whose main aim is to explore the determinants of cardiovascular diseases in individuals aged 35–75 years in Lausanne, Switzerland. The study is representative of the Lausanne area having similar age, gender, and postal code distributions to those of the source population [31]. The second follow-up of the cohort was carried out from 2014 to 2017 and collected physical activity information by accelerometry (see below). Because of this, we only considered cross-sectional data from this second follow-up for analysis.

Only individuals of the second follow-up who participated in the collection of accelerometry data were considered for analysis. Participants were excluded if they did not have valid accelerometry data (see below), lived outside the urban area of Lausanne, or if geolocated data or covariates (see below) were missing.

The institutional Ethics Committee of the University of Lausanne, which afterwards became the Ethics Commission of Canton Vaud (www.cer-vd.ch) approved the baseline CoLaus study (reference 16/03). The approval was renewed for the first (reference 33/09) and the second (reference 26/14) follow-ups. The study was performed in agreement with the Helsinki declaration and its former amendments, and in accordance with the applicable Swiss legislation. All participants gave their signed informed consent before entering the study.

### Physical activity data collection process

A detailed description of the physical activity data collection process was described previously by Gubelmann et al. [32]. Overall, physical activity data were obtained from a wrist-worn triaxial accelerometer (GENEActiv, Activinsights Ltd., United Kingdom) used in the right wrist of the participants at a frequency of 50 Hz for 14 days and converted into 1-minute epoch files using the GENEActiv macros [33]. The GGIR algorithm [34] was then used to transform accelerometry data suitable for analysis. Additionally, we used the PAMPRO methodology [35] as a sensitivity analysis to validate the findings obtained, results are shown in S2 Fig. Data were valid if individuals had accelerometry information ≥10 h during weekdays and ≥8 h during weekends [32]. A minimum of 5 days for weekdays and 2 days for weekends of accelerometry recorded data were required to consider observations valid for analysis [36].

Daily sedentary, light, moderate, and vigorous physical activity intensities were averaged and stratified by weekdays and weekends. MVPA intensities were considered to assess physical activity. MVPA is defined as the sum (in minutes) of the average daily moderate and vigorous physical activity obtained from the accelerometry data. Stratified results for weekdays and weekends are shown in S3 Fig.

### Covariates

Individual data were self-reported and physical measures (i.e. height and weight) were taken by trained professionals in a single visit at the Centre Hospitalier Universitaire Vaudois. Socioeconomic and demographic covariates were age (years), gender, ethnicity (white and non-white), marital status (single, married, divorced, or widowed), education level (low, medium, or high), and work status (low, medium, high, or not working). We also included the annual median household income at the neighborhood level (1 CHF = 1.10 USD, November 2020) obtained from the 2009 Lausanne Census (https://www.vd.ch/themes/etat-droit-finances/statistique) and assigned to the place of residence of each individual. Additionally, we assessed the body mass index (BMI) and the season when MVPA was assessed (spring, summer, autumn, winter).

As factors characterizing the built environment, we considered the number of parks and their location obtained from the Federal Office of Topography (https://www.swisstopo.admin.ch/en/home.html), cycling pathways length and preferential pedestrian zones (pedestrian and meeting places) from the Service des Routes et de la Mobilité (https://www.asitvd.ch/), population density (years 2014–2017) and land use mix area coverage from the Federal Statistical Office (FSO) reported at the hectometric level (https://www.bfs.admin.ch/bfs/en/home.html). Land use mix area coverage was calculated using the formula proposed by Frank et al. [37] and adapted to 5 land use categories reported by the FSO (residential, commercial & industrial, public, recreational, and natural). We also used this formula to calculate the coverage area of each individual land use category. Results range from 0 to 1; 0 stands for no land use mix area coverage and 1 for maximum land use mix area coverage. Likewise, to evaluate public transport accessibility, we calculated the number of public transport stops and the walking time it takes to reach the closest public transport stop from the place of residence of each individual using street network data from © OpenStreetMap contributors (https://www.openstreetmap.org/copyright) and Tobler’s Hiking function [38]. We also used this data source to calculate street connectivity (3 or greater number of streets intersections). All these built environmental variables were assigned to the place of residence of each participant within a buffer of 800 m and, except for land use mix area coverages and walking time to reach the closest public transport stop, the values were divided by the buffer’s area (1.98 km^2^) to standardize the variables into densities.

### Statistical analysis

We calculated Local Moran’s I (LMI) to identify possible spatial clusters of MVPA. LMI measures spatial dependence and highlights local clusters of the variable of interest, here MVPA [39]. This method was derived from the global Moran’s I index that evaluates spatial autocorrelation of a variable across a geographic area and ranges from -1 to 1 [40]. A value of 0 indicates no spatial dependence (observations are randomly distributed in space). A Moran’s scatterplot displays the relationship between the observed MVPA for individual i and the mean MVPA for the individuals located in a specified neighborhood around individual i (the weighted MVPA). Four distinct categories are identified according to the position of individuals in the four quadrants of the Moran’s scatterplot: i) high-high: individuals with a high MVPA surrounded by neighbors showing a high MVPA also, ii) low-low: individuals with a low MVPA surrounded by neighbors with a low MVPA also, iii) low-high: individuals with low MVPA surrounded by neighbors with a high MVPA (considered as outliers), iv) high-low: individuals with a high MVPA and surrounded by neighbors with low a MVPA (outliers also). There is an additional fifth category corresponding to individuals which are randomly distributed in space. The latter category is determined using a significance test based on Monte-Carlo random permutations of MVPA observations [39]. It is calculated as (M+1) / (P+1); P corresponds to the number of permutations and M to the number of instances where a permutation statistic is greater or equal than the observed value (if positive LMI), or lower or equal than the observed value (if negative LMI). In this study, we tested 999 Monte-Carlo permutations and an α level of 0.05, the spatial lag used was 800 m [41]. LMI was calculated on raw MVPA and on the residuals of an MVPA model adjusted for socioeconomic and demographic factors (age, neighborhood household income, gender, civil status, ethnicity, job status, BMI, and seasonality) using a linear median -50^th^ quantile- regression (S1 Appendix). This method is recommended over the traditional ordinary least squares regression when the data does not follow a normal distribution [42]. Moreover, MVPA was transformed to its square root for statistical analysis and was back-transformed to its real value for the description of the results. We also ran LMI using different spatial lags of 400, 600, 1000, and 1200 m, and the analyses showed similar spatial patterns to the ones we obtained with 800m (S4 Fig).

Due to the low number of individuals contained within the spatial clusters observed, statistical comparisons were carried out using non-parametric methods (significance threshold of p<0.05); chi-square or fisher tests (when N < 5) for categorical data, and Kruskal Wallis tests (including Bonferroni-Holm’s correction) for numeric variables. Because of the above, data are reported as median and interquartile range (IQR) for numeric variables and frequencies and percentages (%) for categorical information.

Moreover, we performed a spatial error model to further assess the spatial association between the built environment with MVPA (S2 Appendix). Briefly, this method models spatial autocorrelation considering an error term (*u*) and a spatial lagged error term (*Wu*) (i.e. the error of a neighbor is affected by the error of a given observation) [43].

We used this spatial error model over other spatial regression methods, such as spatial lag and spatial autoregressive moving average models (information not shown) because the former was the model better fitting the data (Akaike information criterion: 16275 vs 16276 and 16278, respectively). Additionally, we removed the variables related to the density of public transport stops, preferential pedestrian areas, cycling pathways, interconnected streets, population density, land use areas, and residential and natural area coverages due to multicollinearity issues (variance inflation factor >5).

Analyses were performed in R version 3.6.3 [44]. Additionally, the rgeoda library [45] was used to run spatial analyses, sf [46] to calculate densities and lengths in spatial buffers for each individual. Maps were drawn with ggplot2 [47].

## Results

The initial sample consisted of 2,967 individuals that participated in the collection of accelerometry data. Among them, 945 individuals living outside the urban area of Lausanne and without valid accelerometry and geolocation data were removed, leading to a dataset of 2,022 observations (68%). A further 133 (6%) observations had missing data and were also removed, leading to a final sample of 1,889 individuals.

Individual variables and characteristics on the built environment of the studied and removed individuals are described in S1 Table. Overall, age was higher in the studied population (63 vs 60), there was a higher proportion of married (56% vs 53%), and a lower proportion of widowed (7% vs 11%) and low education rates (52% vs 59%) in the individuals included in the analysis. No statistically significant differences were observed in the built environment between included and excluded participants. The median age of the studied individuals was 63 years (IQR: 16) and there were 1,049 (55%) women. The median daily MVPA was 20.3 mins (IQR: 23.0).

### Spatial clusters of raw MVPA and adjusted MVPA

**Fig 1:**
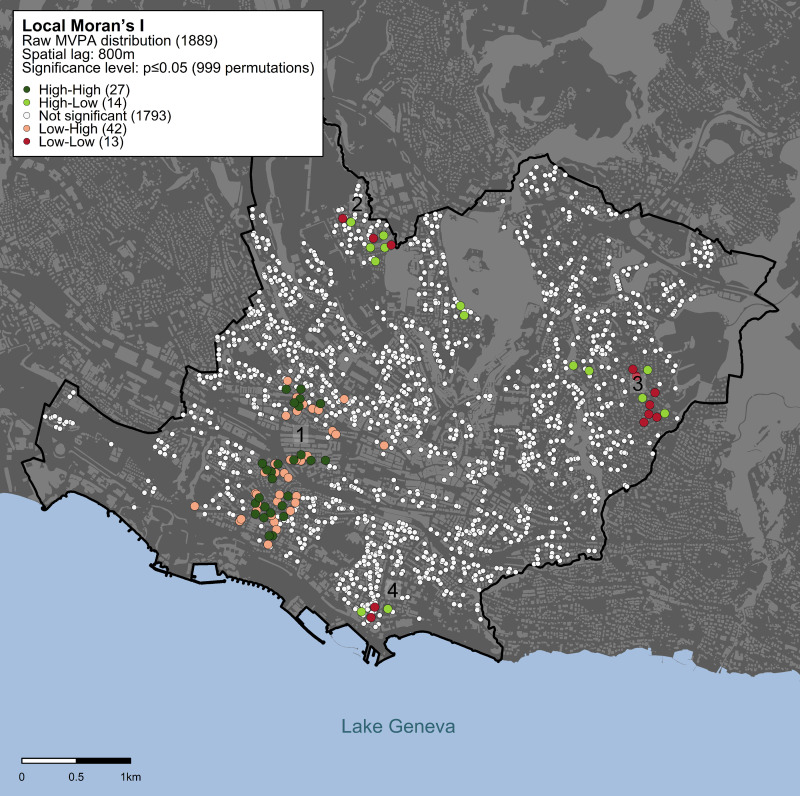
Spatial clusters of raw MVPA using Local Moran’s I statistics. Statistical significance is assessed based on an α threshold of p<0.05 within a spatial lag of 800 m. Dark-green dots indicate individuals with high MVPA values surrounded by neighbors also showing high MVPA values; red dots indicate individuals with low MVPA surrounded by neighbors with low MVPA values; light-green dots indicate individuals with high MVPA values surrounded by neighbors showing low MVPA values; pink dots indicate individuals with low MVPA values surrounded by neighbors with high MVPA values; white dots indicate individuals whose MVPA values are randomly distributed in the geographic space. Landmarks (1–4) are shown to facilitate the description and interpretation of the results. Map was created using data from the Swiss Federal Office of Topography (swisstopo).

We detected spatial clusters of raw MVPA in the urban area of Lausanne (Fig 1). We identified one small, but statistically significant, concentration of individuals (1%) in clusters of high MVPA and a concentration of 2% of participants with discordant MVPA levels (Low-High) in the south-western region of the urban area (landmark #1). A small concentration (1%) of participants in low MVPA clusters and individuals having intertwined values of MVPA (High-Low) were observed in the north-western, eastern, and southern regions (landmarks #2–4).

**Fig 2:**
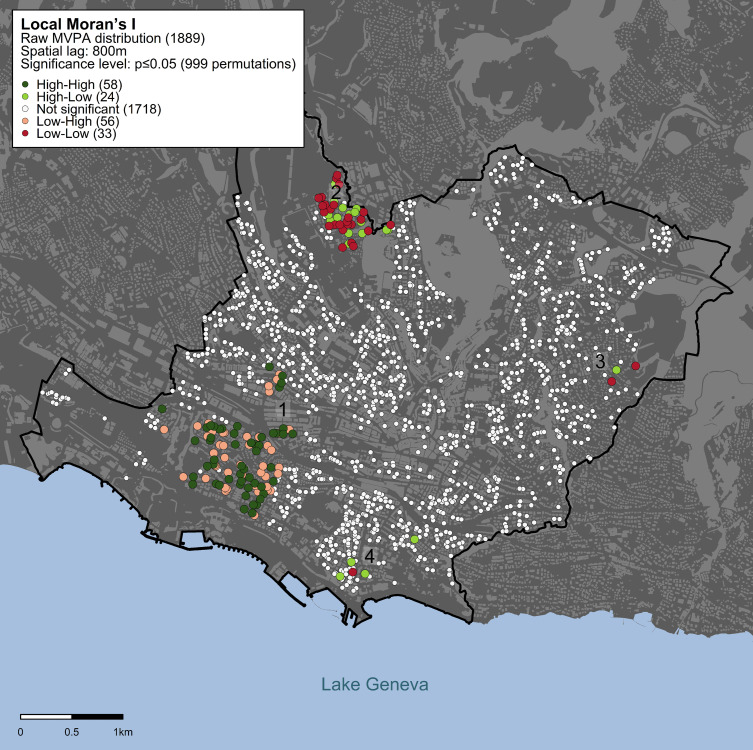
Spatial clusters of MVPA adjusted for socioeconomic and demographic factors using Local Moran’s I statistics. Statistical significance is assessed based on an α threshold of p<0.05 within a spatial lag of 800 m. Dark-green dots indicate individuals with high MVPA values surrounded by neighbors also showing high MVPA values; red dots indicate individuals with low MVPA surrounded by neighbors with low MVPA values; light-green dots indicate individuals with high MVPA values surrounded by neighbors showing low MVPA values; pink dots indicate individuals with low MVPA values surrounded by neighbors with high MVPA values; white dots indicate individuals whose MVPA values are randomly distributed in the geographic space. Landmarks (1–4) are shown to facilitate the description and interpretation of the result. Map was created using data from the Swiss Federal Office of Topography (swisstopo).

Spatial clusters of MVPA adjusted for socioeconomic and demographic factors (Fig 2) were geographically distributed similarly to the clusters of raw MVPA (Fig 1). However, there was a higher concentration of Low-Low and High-Low clusters in the north-western area (landmark #2) and a lower concentration in the eastern and southern regions (landmarks #3 and #4). The overall size of low-low clusters increased from 1% to 2% of the studied population. High-High clusters increased their size from 1% to 3% (landmark #1).

### Built environment characteristics in raw MVPA spatial clusters

Statistically significant built environment differences (p<0.05) were observed among spatial clusters of raw MVPA (Table 1). Clusters with high levels of MVPA (High-High) were located in areas with a higher density of parks (high: 2 *vs* low: 0), public transport stops (high: 28 *vs* low: 11), preferential pedestrian areas (high: 3 *vs* low: 2), cycling pathways (high: 6.7 *vs* low: 1.8 km), interconnected streets (high: 133 *vs* low: 55), population density (high: 11,708 *vs* low: 5,061), and public (high: 0.28 *vs* low: 0.21), and commercial & industrial land use areas (high: 0.18 *vs* low: 0.03) compared to clusters of low MVPA (Low-Low). In contrast, clusters of low MVPA were in locations with a higher presence of natural areas (low: 0.25 *vs* high: 0). Socioeconomic characteristics of individuals among clusters can be observed in S2 Table; older participants and the proportion of non-workers were statistically higher in low MVPA clusters.

**Table 1:** Built environment characteristics in raw MVPA spatial clusters.

Built environment characteristics	No spatial dependence	High-High	Low-Low	High-Low	Low-High	p-value^a^
N	1793 (95%)	27 (1%)	13 (1%)	14 (1%)	42 (2%)	
MVPA (mins)	20.3 (23.2)	35.2 (29.9)	16.5 (9.8)	37.8 (26.1)	12.1 (10.0)	<0.001^b^
Density of parks	2 (2)	2 (1)	0 (1)	0 (1)	2 (1)	<0.001^c^
Density of public transport stops	20 (10)	28 (5)	11 (4)	16 (4)	27 (6)	<0.001^c^
Walking time to closest public transport stop (mins)	6.1 (4.1)	6.2 (3.0)	7.2 (7.3)	7.8 (5.5)	6.1 (5.6)	0.22
Density of preferential pedestrian areas	3 (6)	3 (1)	2 (1)	2 (2)	3 (2)	0.001^c^
Density of cycling path length (km)	4.2 (3.1)	6.7 (0.7)	1.8 (1.8)	3.1 (0.6)	6.7 (0.6)	<0.001^c^
Density of interconnected streets >3	110 (63)	133 (50)	55 (23)	73 (24)	133 (57)	<0.001^c^
Population density	8838 (4933)	11708 (5655)	5061 (1831)	7016 (2004)	12365 (6349)	<0.001^d^
Land use mix area coverage	0.69 (0.11)	0.72 (0.05)	0.46 (0.39)	0.78 (0.29)	0.71 (0.06)	0.26
Residential area coverage	0.33 (0.06)	0.33 (0.05)	0.20 (0.17)	0.36 (0.12)	0.33 (0.05)	0.45
Commercial & industrial area coverage	0.07 (0.08)	0.18 (0.03)	0.03 (0.13)	0.12 (0.10)	0.18 (0.04)	<0.001^c^
Public places area coverage	0.27 (0.08)	0.28 (0.02)	0.21 (0.01)	0.22 (0.04)	0.28 (0.02)	<0.001^c^
Recreational area coverage	0.31 (0.11)	0.32 (0.06)	0.09 (0.28)	0.28 (0.23)	0.33 (0.06)	0.02^e^
Natural area coverage	0.15 (0.24)	0 (0.04)	0.25 (0.09)	0.30 (0.09)	0 (0.04)	<0.001^c^

Except for N, variables are described using the median (IQR).

^a^ p-values for High-High *vs* High-Low *vs* Low-High *vs* Low-low

^b^ High-High and High-Low were statistically different from Low-Low and Low-High clusters.

^c^ High-High and Low-High were statistically different from Low-Low and High-Low clusters.

^d^ High-High and Low-High were statistically different from Low-Low and High-Low clusters, but also High-Low statistically different from Low-Low clusters.

^e^ Low-High was statistically different from Low-Low clusters.

### Built environment characteristics in MVPA spatial clusters after adjustment for socioeconomic and demographic factors

The characteristics of the built environment among spatial clusters of MVPA adjusted for socioeconomic and demographic factors (Table 2) were similar to the raw MVPA model. Clusters of high MVPA (High-High) presented a higher density of parks (high: 2 *vs* low: 0), public transport stops (high: 22 *vs* low: 12), preferential pedestrian areas (high: 3 *vs* low: 1), cycling pathways (high: 6.7 *vs* low: 2.6 km), interconnected streets (high: 128 *vs* low: 53), population density (high: 8,886 *vs* low: 5,874), and public (high: 0.27 *vs* low: 0.21), and commercial & industrial land use areas (high: 0.20 *vs* low: 0.14) than clusters of low MVPA (Low-Low). High MVPA clusters were also located in areas where the amount of walking time to reach the closest public transport stops was shorter than low MVPA (high 5.8 *vs* low: 8.9 mins). Likewise, clusters of low MVPA showed more natural (low: 0.32 *vs* high: 0) and residential areas (low: 0.37 *vs* high: 0.35) and land use mix area coverage (low: 0.86 *vs* high: 0.73). Socioeconomic and demographic characteristics are described in S3 Table; neighborhood household income was statistically significantly lower in low MVPA clusters.

**Table 2:** Built environment characteristics in spatial clusters of MVPA adjusted for socioeconomic and demographic factors.

Built environment characteristics	No spatial dependence	High-High	Low-Low	High-Low	Low-High	p-value^a^
N	1718 (91%)	58 (3%)	33 (2%)	24 (1%)	56 (3%)	
MVPA (mins)	20.3 (22.9)	38.5 (34.6)	10.9 (14.3)	38.6 (23.5)	10.3 (12.0)	<0.001^b^
Density of parks	2 (2)	2 (1)	0 (1)	0 (1)	1 (1)	<0.001^c^
Density of public transport stops	20 (16)	22 (8)	12 (6)	13 (3)	23 (6)	<0.001^c^
Walking time to closest public transport stop (mins)	6.0 (4.0)	5.8 (4.1)	8.9 (5.1)	8.9 (3.2)	6.1 (4.1)	<0.001^d^
Density of preferential pedestrian areas	3 (6)	3 (1)	1 (1)	1 (1)	3 (1)	<0.001^c^
Density of cycling path length (km)	4.2 (2.9)	6.7 (0.7)	2.6 (0.7)	2.7 (0.4)	6.5 (0.6)	<0.001^c^
Density of interconnected streets >3	111 (63)	128 (28)	53 (16)	54 (17)	127 (27)	<0.001^c^
Population density	8955 (4966)	8986 (4535)	5874 (1490)	5949 (1483)	8974 (3716)	<0.001^c^
Land use mix area coverage	0.69 (0.11)	0.73 (0.03)	0.86 (0.03)	0.86 (0.03)	0.74 (0.03)	<0.001^c^
Residential area coverage	0.33 (0.06)	0.35 (0.04)	0.37 (0.01)	0.37 (0.01)	0.36 (0.03)	<0.001^c^
Commercial & industrial area coverage	0.06 (0.08)	0.20 (0.05)	0.14 (0.02)	0.14 (0.03)	0.20 (0.06)	<0.001^c^
Public places area coverage	0.28 (0.08)	0.27 (0.02)	0.21 (0.03)	0.22 (0.04)	0.27 (0.02)	<0.001^c^
Recreational area coverage	0.31 (0.11)	0.36 (0.04)	0.34 (0.02)	0.035 (0.02)	0.36 (0.03)	0.002^e^
Natural area coverage	0.15 (0.22)	0 (0.04)	0.32 (0.02)	0.33 (0.04)	0 (0.04)	<0.001^c^

Except for N, variables are described using the median (IQR).

^a^ p-values for High-High *vs* High-Low *vs* Low-High *vs* Low-low

^b^ High-High and High-Low were statistically different from Low-Low and Low-High clusters.

^c^ High-High and Low-High were statistically different from Low-Low and High-Low clusters.

^d^ High-High and Low-High were statistically different from Low-Low, High-Low statistically different from High-High but not Low-High clusters.

^e^ High-High and Low-High were statistically different from Low-Low but not High-Low clusters.

### Spatial error model

The spatial error model (S2 Appendix) showed that older individuals and participants having higher BMI, education level, and job position were associated with lower levels of MVPA. On the contrary, a wider presence of commercial & industrial areas and a lower walking distance to public transport were spatially associated with higher levels of MVPA. The density of parks, and recreation and public areas coverages did not show any statistically significant association.

## Discussion

We observed local geographic clusters of high and low MVPA in the urban area of Lausanne. Despite the low number of individuals within clusters, we identified clear differences in the composition of the built environment among clusters, showing that there is a particular underlying composition of the built environment in high MVPA clusters, and a clearly different composition of the built environment in low MVPA clusters. Spatial clusters of high MVPA were located in zones with a higher population density, a better accessibility to parks and to public transportation, more cycling pathways, preferential pedestrian zones, interconnected streets, and public, commercial & industrial zones. As an example, the larger high MVPA cluster (landmark #1 on Fig 1) was clearly located right next to a recreation area equipped with sports facilities on the shores of the lake. The geographic distribution of MVPA was similar for the raw MVPA model and the model adjusted for socioeconomic and demographic factors.

Despite socioeconomic status has been associated as a mediator in the association of the built environment and physical activity [48, 49], we did not find clear socioeconomic and demographic differences among clusters of low and high MVPA. However, we did highlight an older population and a higher proportion of non-workers in low raw MVPA clusters, and a lower neighborhood household income in low MVPA clusters after adjustment for socioeconomic and demographic factors. Huang et al. [26], Tamura et al. [24], and Valson et al. [28] also observed inconsistent findings related to socioeconomic determinants and spatial clusters of physical activity. These inconsistencies may be related to our lack of adjustment for social conditions, such as crime rates, pedestrian safety, and harmful land uses [50].

Studies that have explored the association of the built environment with physical activity from a geographic perspective have found mixed evidence [24, 25, 27]. However, there is an overall agreement that environments that favor walkability, such as higher population density and street connectivity, are associated with higher physical activity levels [23, 24, 26, 28]. In contrast, we did not find a positive association between a higher land use area coverage with the spatial clusters of high physical activity [23, 24, 28]. This is probably because we were unable to stratify by different types of commercial & industrial land use areas such as food, retails, services, cultural, and physical activity as proposed by Frank et al. [37].

The association between spatial clusters of physical activity and green areas is debatable, with no clear positive association [25, 28]. We found that clusters with high MVPA exhibited a higher density of parks and recreational areas but not natural areas. This may highlight important considerations when measuring greenness. As natural areas are usually located at the boundaries of the urban perimeter, the presence of greener areas may not necessarily imply they are adequate or accessible for leisure physical activity or active transportation due to long commuting distances to the destination of individuals [51, 52]. Additionally, we hypothesized that city dwellers need a minimum of urban infrastructure or of motivation (i.e. shopping) to move, and that they are not attracted by these natural areas despite their obvious health benefits.

In addition to the above built environmental factors, we also included other determinants, which to our knowledge, are not typically used when analyzing the association between the built environment and spatial clusters of physical activity. We observed that clusters with high MVPA values were located in areas with higher accessibility to cycling pathways and areas that favor pedestrians. Such findings are consistent with other studies not following a spatial methodology [7, 51, 53]. Similarly, as found in our study, higher accessibility to public transport has also been associated to higher levels of physical activity [7].

The spatial error regression model showed some expected associations of the built environment with higher levels of MVPA such as a higher presence of commercial & industrial areas and lower walking distance to public transport, which is in line with the observed in the Moran’s I analysis. However, we did not observe any association with the density of parks and recreation and public area coverages. This evidence some inconclusive associations of MVPA with the built environment also observed in other studies using a spatial approach [21, 22, 24, 25, 28], and may suggest some characteristics of the built environment only influence active physical behaviors locally and not in the entire urban area.

Geographic patterns of MVPA on weekdays were similar to the patterns on weekends. However, we observed a higher concentration of individuals in MVPA clusters during weekdays, which may highlight different physical activity interactions of individuals with their built environment depending on the day of the week [54, 55]. The comparison of the GGIR algorithm with the PAMPRO methodology to calculate MVPA showed some similarities but also some differences in their MVPA spatial distribution. We decided to use GGIR, but certainly, we could also have chosen PAMPRO for our main analysis. Further studies are needed to validate which method is best suited to measure physical activity using accelerometry data.

Interestingly, we observed similarities between the geographic distribution of MVPA and the distribution of BMI observed in a previous study of the same urban area [41]: one cluster of low MVPA overlaps a cluster of high BMI and a cluster of high MVPA overlaps a cluster of low BMI. This finding is aligned with another study that has found overlapping clusters of BMI and physical activity [56] and could be of great help as a measure of the success of policies promoting healthier built environments.

### Limitations

Our study has several limitations. First, we were not able to assess the working environment nor to track the exact location where people performed physical activity, which would be useful to precisely pinpoint what type of built environment characterized best areas where physical activity was performed [57, 58], although Holliday et al. [58] and Cohen et al. [59] report that most physical activity is performed at home and near locations, such as green areas located <1 mile away from the residence of participants. Second, since this is an observational study, we may not be able to assess correctly a dynamic changing environment such as the built environment and our findings may suffer from reverse causality, (i.e. individuals may select their place of residence based on neighborhood characteristics that favor their lifestyles) [12]. However, evidence shows that the built environment influences physical activity independently of residential self-selection [13], and except for population density, we used built environment variables that are unlikely to have large modifications over time in the urban area of Lausanne (i.e. land use area coverages, interconnected streets, location of public transport stops and parks, etc.). This cross-sectional approach also limits the assessment of health behavior changes from the start of the cohort, nevertheless, there is not yet clear evidence that participating in a study influences changes in individuals’ behaviors [60, 61]. Third, we observed few individuals belonging to spatial clusters of PA resulting in limited statistical power and making our results not representative of the entire city but only of some local areas. Local findings, that may turn in local neighborhood policies, may help to improve health and reduce inequalities at a city scale [62]. Fourth, we did not differentiate between transportation and recreational MVPA, and such a distinction could have been of interest to identify how the built environment affects different practices of physical activity [63]. Fifth, our results may not be entirely generalizable to other locations as built environment characteristics may differ.

### Strengths

To the best of our knowledge, this is the first study that assesses spatial dependence of objectively measured MVPA using individual geolocated data, making our study less prone to bias [37, 64]. We used additional variables (i.e. accessibility to cycling pathways, pedestrian areas, and public transport) that are not usually assessed in the study of spatial patterns of physical activity and the built environment, and we considered multiple dimensions of the neighborhood environment [65]. We showed that this inclusion brings a new perspective and evidence for such association. Additionally, we assessed individual spatial clusters of MVPA, which allowed us to evaluate the spatial dependence of MVPA on a local scale.

### Impact for public health policy

Our findings highlight the utility of spatial analysis to explore the influence the characteristics of the built environment have on physical activity and to identify populations at risk, which could be useful for the development of public health policies related to urban planning. Evidence on the success of initiatives modifying the built environment infrastructure to improve physical activity levels is promising [66]. Therefore, policymakers should be encouraged to favor the development of public built environment interventions that promote healthier behaviors -and indirectly more ecological cities-, such as increasing the availability of parks and cycling pathways and facilitating access to public transport and pedestrian areas.

### Conclusions

Although with a low number of individuals, geographic clusters of high MVPA were detected in urban areas where specific local compositions of the built environment favor physical activity. Adjustment for socioeconomic and demographic factors did not impact the geographic patterns or built environment characteristics of the clusters. Favoring the planning of urban environments promoting physical activity and active lifestyles should be considered when developing public policies.

## Supporting information

S1 Fig Built environmental characteristics of the Lausanne urban area. (DOCX)

S2 Fig Spatial clusters of raw MVPA using Local Moran’s I statistics and the PAMPRO algorithm. (DOCX)

S3 Fig Spatial clusters of raw MVPA for weekdays (a) and weekends (b) using Local Moran’s I statistics.
(DOCX)

S4 Fig Spatial clusters of raw MVPA within spatial lags of 400 (a), 600 (b), 1000 (c), and 1200 m (d) using Local Moran’s I statistics.
(DOCX)

S1 Appendix Regression results of MVPA adjusted for socioeconomic and demographic factors. (DOCX)

S2 Appendix Spatial error model results of MVPA adjusted for socioeconomic, demographic, and built environment factors. (DOCX)

S1 Table Comparison of population and built environment characteristics between included and removed individuals. (DOCX)

S2 Table Population socioeconomic and demographic characteristics of raw MVPA spatial clusters. (DOCX)

S3 Table Population socioeconomic and demographic characteristics of adjusted MVPA spatial clusters. (DOCX)
