# Supplementary material for: Geographic clusters of objectively measured physical activity and the characteristics of their built environment in a Swiss urban area
Source: PLoS One. 2022 Feb 23;17(2):e0252255. doi: 10.1371/journal.pone.0252255 (PMC8865698; doi:10.1371/journal.pone.0252255)
Supplement: S1 Fig — (DOCX) [file pone.0252255.s001.docx]

**Urban area of Lausanne**

**S1 Fig. Built environmental characteristics of the Lausanne urban area.** Map was created using data from the Swiss Federal Office of Topography (swisstopo).

**
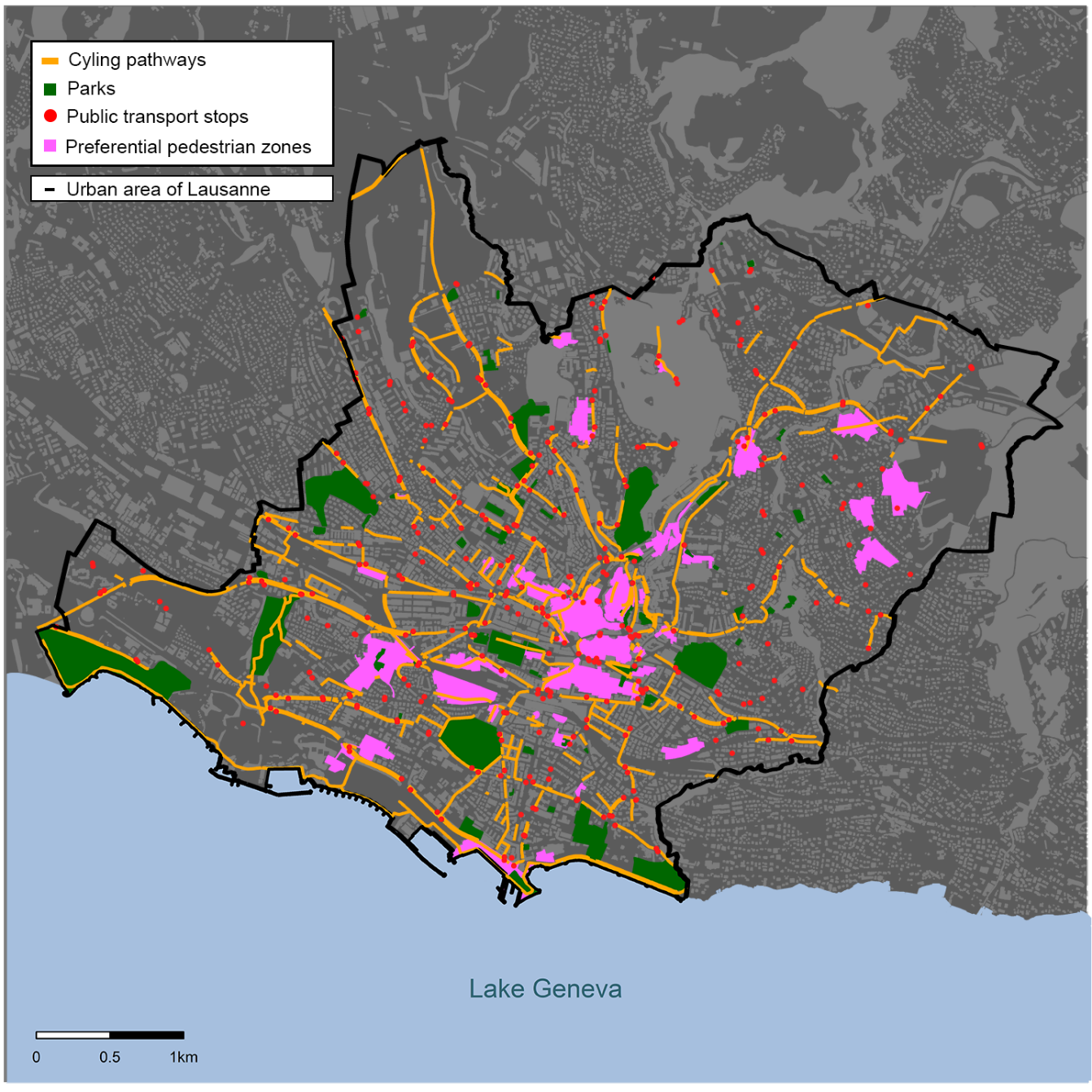
**
