# Supplementary material for: Geographic clusters of objectively measured physical activity and the characteristics of their built environment in a Swiss urban area
Source: PLoS One. 2022 Feb 23;17(2):e0252255. doi: 10.1371/journal.pone.0252255 (PMC8865698; doi:10.1371/journal.pone.0252255)
Supplement: S2 Fig — (DOCX) [file pone.0252255.s002.docx]

**Spatial distribution of raw MVPA (PAMPRO algorithm)**

**S2 Fig. Spatial clusters of raw MVPA using Local Moran’s I statistics and the PAMPRO algorithm.** Statistical significance is assessed based on an α threshold of p<0.05 within a spatial lag of 800 m. Dark-green dots indicate individuals with high MVPA values surrounded by neighbors also showing high MVPA values; red dots indicate individuals with low MVPA surrounded by neighbors with low MVPA values; light-green dots indicate individuals with high MVPA values surrounded by neighbors showing low MVPA values; pink dots indicate individuals with low MVPA values surrounded by neighbors with high MVPA values; white dots indicate individuals whose MVPA values are randomly distributed in the geographic space. Landmarks (1-4) are shown to facilitate the description and interpretation of the results. Map was created using data from the Swiss Federal Office of Topography (swisstopo).

**
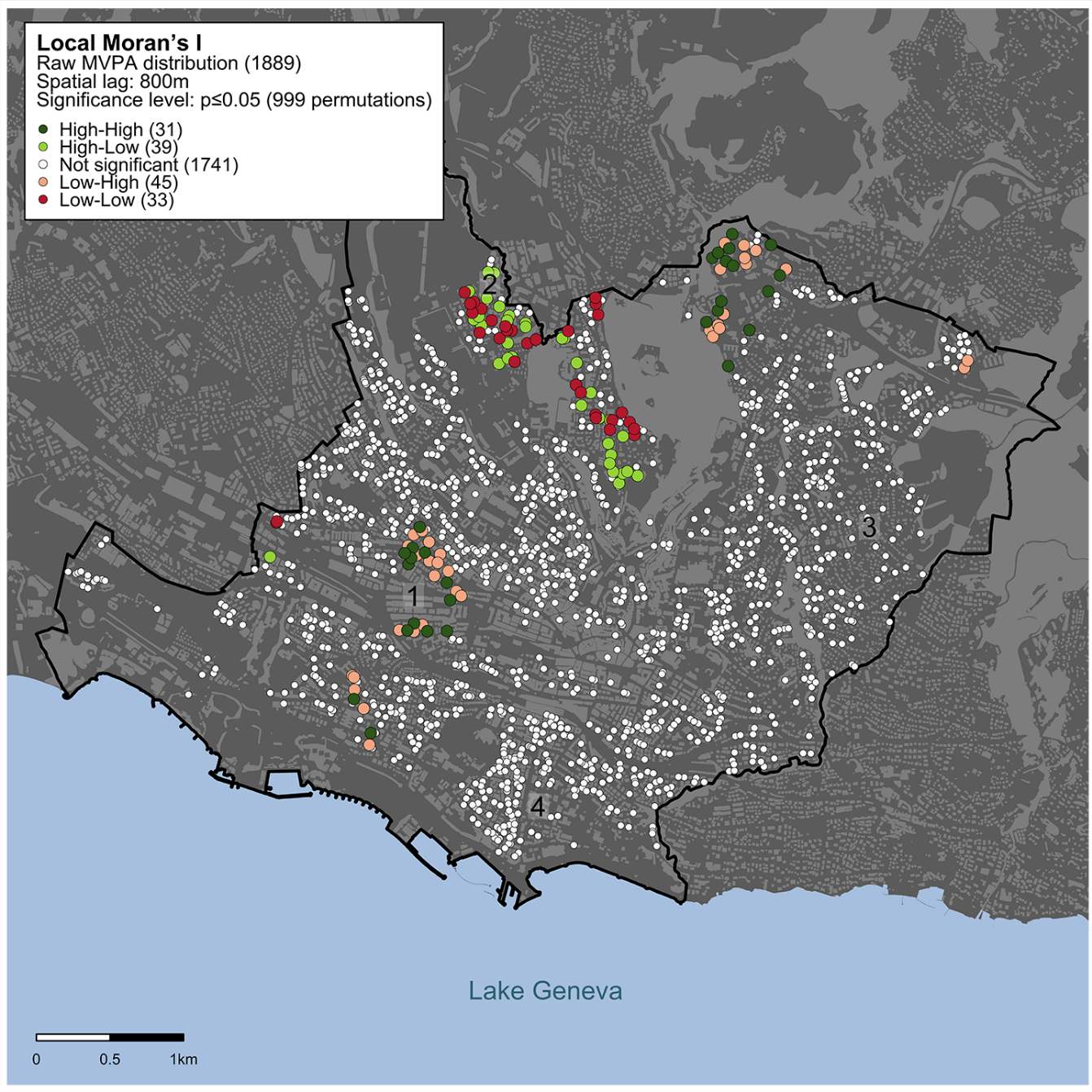
**
