# Supplementary material for: Geographic clusters of objectively measured physical activity and the characteristics of their built environment in a Swiss urban area
Source: PLoS One. 2022 Feb 23;17(2):e0252255. doi: 10.1371/journal.pone.0252255 (PMC8865698; doi:10.1371/journal.pone.0252255)
Supplement: S1 Appendix — (DOCX) [file pone.0252255.s005.docx]

**S1 Appendix. Regression results of MVPA adjusted for socioeconomic and demographic factors.**

| Variable | Coefficient | Confidence Intervals (95%) | Standard Error | P-value |
| --- | --- | --- | --- | --- |
| Intercept | 11.86 | 11.09, 12.73 | 0.56 | <0.001 |
| Age (years) | -0.08 | -0.09, -0.07 | 0.01 | <0.001 |
| Neighborhood household income (USD) | 0.0001 | -0.0001, 0.0001 | 0.0001 | 0.72 |
| BMI (kg/m^2^) | -0.08 | -0.10, -0.06 | 0.01 | <0.001 |
| Men vs women | -0.09 | -0.23, 0.10 | 0.11 | 0.42 |
| Medium vs low education level | -0.26 | -0.44, -0.09 | 0.11 | 0.02 |
| High vs low education level | -0.24 | -0.44, -0.02 | 0.13 | 0.05 |
| Married vs single | 0.10 | -0.24, 0.35 | 0.17 | 0.55 |
| Divorced vs single | 0.01 | -0.33, 0.23 | 0.17 | 0.94 |
| Widowed vs single | -0.40 | -0.76, -0.08 | 0.23 | 0.04 |
| White vs non-white | -0.01 | -0.30, 0.24 | 0.19 | 0.98 |
| Medium vs low job status | -0.12 | -0.50, 0.07 | 0.16 | 0.45 |
| High vs low job status | -0.46 | -0.75, -0.11 | 0.22 | 0.03 |
| Not working vs low job status | -0.32 | -0.68, -0.10 | 0.16 | 0.05 |
| Summer vs spring | 0.08 | -0.8, 0.34 | 0.14 | 0.56 |
| Summer vs autumn | 0.06 | -0.11, 0.27 | 0.13 | 0.62 |
| Summer vs winter | -0.20 | -0.71, 0.08 | 0.14 | 0.13 |
