# Supplementary material for: Geographic clusters of objectively measured physical activity and the characteristics of their built environment in a Swiss urban area
Source: PLoS One. 2022 Feb 23;17(2):e0252255. doi: 10.1371/journal.pone.0252255 (PMC8865698; doi:10.1371/journal.pone.0252255)
Supplement: S2 Appendix — (DOCX) [file pone.0252255.s006.docx]

**S2 Appendix. Spatial error model results of MVPA adjusted for socioeconomic, demographic, and built environment factors.**

| Variable | Coefficient | Confidence Intervals (95%) | Standard Error | P-value |
| --- | --- | --- | --- | --- |
| Intercept | 9.55 | (8.44, 10.65) | 5.62 | <0.001 |
| Age (years) | -0.72 | (-0.83, -0.61) | 0.05 | <0.001 |
| Neighborhood household income (USD) | 0.004 | (-0.004, 0.001) | 0.0004 | 0.34 |
| BMI (kg/m^2^) | -0.85 | (-1.03, -0.07) | 0.09 | <0.001 |
| Men vs women | 1.54 | (-0.18, 3.25) | 0.88 | 0.07 |
| Medium vs low education level | -4.45 | (-6.43, -2.46) | 1.01 | <0.001 |
| High vs low education level | -4.76 | (-7.11, -2.40) | 1.20 | <0.001 |
| Married vs single | 2.34 | (-0.04, 4.71) | 1.21 | 0.06 |
| Divorced vs single | 2.75 | (-2.43, 2.98) | 1.38 | 0.84 |
| Widowed vs single | -0.11 | (-3.99, 3.77) | 1.98 | 0.95 |
| White vs non-white | 1.05 | (-2.06, 4.16) | 1.58 | 0.51 |
| Medium vs low job status | -3.02 | (-5.69, -0.35) | 1.36 | 0.02 |
| High vs low job status | -7.18 | (-10.60, -3.77) | 1.74 | <0.001 |
| Not working vs low job status | -5.62 | (-8.12, -3.11) | 1.28 | <0.001 |
| Summer vs spring | -0.44 | (-2.74, 1.85) | 1.17 | 0.70 |
| Summer vs autumn | -0.31 | (-2.52, 1.88) | 1.12 | 0.78 |
| Summer vs winter | -1.91 | (-4.19, 0.37) | 1.16 | 0.10 |
| Density of parks | 0.29 | (-0.63, 1.23) | 0.47 | 0.53 |
| Walking time to closest public transport stop (mins) | -0.20 | (-0.43, 0.00) | 0.11 | 0.05 |
| Commercial **&** industrial area coverage | 1.31 | (0.48, 2.58) | 0.64 | 0.04 |
| Public places area coverage | 1.35 | (-18.19, 20.89) | 9.97 | 0.89 |
| Recreational area coverage | -3.56 | (-14.53, 7.39) | 5.59 | 0.52 |
