# Supplementary material for: Geographic clusters of objectively measured physical activity and the characteristics of their built environment in a Swiss urban area
Source: PLoS One. 2022 Feb 23;17(2):e0252255. doi: 10.1371/journal.pone.0252255 (PMC8865698; doi:10.1371/journal.pone.0252255)
Supplement: S3 Table — (DOCX) [file pone.0252255.s009.docx]

**S3** **Table. Population socioeconomic and demographic characteristics of adjusted MVPA spatial clusters.**

| Socioeconomic characteristics | No spatial dependence | High-High | Low-Low | High-Low | Low-High | p-value^a^ |
| --- | --- | --- | --- | --- | --- | --- |
|  | Median (IQR) or  Frequencies (%) | Median (IQR) or  Frequencies (%) | Median (IQR) or  Frequencies (%) | Median (IQR) or  Frequencies (%) | Median (IQR) or  Frequencies (%) |  |
| N | 1718 (91%) | 58 (3%) | 33 (2%) | 24 (1%) | 56 (3%) |  |
| Age (years) | 63 (16) | 62 (21) | 68 (19) | 66 (19) | 64 (18) | 0.88 |
| Neighborhood household income (USD) | 56253 (12309) | 56705 (12368) | 45774 (0) | 45774 (0) | 56705 (12368) | 0.001 |
| BMI (kg/m^2^) | 25.9 (5.9) | 25.7 (6.9) | 25.7 (3.8) | 25.8 (5.2) | 25.9 (5.2) | 0.96 |
| Women | 948 (55%) | 33 (60%) | 20 (61%) | 12 (50%) | 36 (64%) | 0.66 |
| Civil status |  |  |  |  |  | 0.22 |
| Single | 263 (15%) | 8 (14%) | 4 (12%) | 3 (12%) | 4 (7%) |  |
| Married | 966 (57%) | 29 (50%) | 16 (49%) | 17 (72%) | 41 (73%) |  |
| Divorced | 379 (22%) | 14 (24%) | 10 (30%) | 3 (12%) | 6 (11%) |  |
| Widowed | 110 (6%) | 7 (12%) | 3 (9%) | 1 (4%) | 5 (9%) |  |
| White | 1584 (92%) | 55 (95%) | 29 (88%) | 21 (88%) | 53 (95%) | 0.42 |
| Education |  |  |  |  |  | 0.67 |
| Low | 887 (52%) | 35 (60%) | 20 (61%) | 14 (59%) | 30 (54%) |  |
| Medium | 455 (26%) | 11 (19%) | 8 (24%) | 8 (33%) | 13 (23%) |  |
| High | 376 (22%) | 12 (21%) | 5 (15%) | 2 (8%) | 13 (23%) |  |
| Job status |  |  |  |  |  | 0.82 |
| Low | 396 (23%) | 16 (28%) | 6 (18%) | 8 (33%) | 12 (21%) |  |
| Medium | 296 (17%) | 9 (15%) | 5 (15%) | 4 (17%) | 8 (14%) |  |
| High | 174 (10%) | 6 (10%) | 1 (3%) | 1 (4%) | 6 (11%) |  |
| Not working | 852 (50%) | 27 (47%) | 21 (64%) | 11 (46%) | 30 (54%) |  |
| Season |  |  |  |  |  | 0.98 |
| Spring | 443 (26%) | 16 (28%) | 10 (30%) | 7 (29%) | 17 (31%) |  |
| Summer | 400 (23%) | 11 (19%) | 6 (18%) | 6 (25%) | 12 (21%) |  |
| Autumn | 471 (27%) | 13 (22%) | 10 (30%) | 5 (21%) | 14 (25%) |  |
| Winter | 404 (24%) | 18 (31%) | 7 (22%) | 6 (25%) | 13 (23%) |  |

^a^p-values for High-High *vs* High-Low *vs* Low-High *vs* Low-low
